# Challenges and opportunities for diagnosis and treatment of rheumatoid arthritis in Latin America

**DOI:** 10.1007/s10067-015-3019-6

**Published:** 2015-07-17

**Authors:** Carlos Pineda, Carlo V Caballero-Uribe

**Affiliations:** Instituto Nacional de Rehabilitación Luis Guillermo Ibarra Ibarra, Calzada México-Xochimlco 289, Colonia Arenal de Guadalupe, Mexico City, 14389 Mexico; Universidad del Norte, Barranquilla, Colombia

Latin America (LA) is a multicultural region, with an estimate of just over 600 million inhabitants, displaying great heterogeneity among their nations. LA countries exhibit complex demographic characteristics because of the interaction of factors such as the different ethnic background of its population, a history of colonialism, and varying immigration patterns, resulting in a diversified population that differs in every country within the region [1].

The LA region has experienced a health transition characterized by fast and complex epidemiological changes in the past decades, combining increasing rates of non-communicable diseases and injuries, while keeping uncontrolled many existing endemic and emerging diseases.

The national health care systems within the LA area are heterogeneous in their organizational structure and complex in their operational configuration, and in the principles guiding the public and private sector roles in the provision of health care services. In LA, medical attention for rheumatoid arthritis (RA) faces a dual challenge, on one side, competes with a budget designed to fight poverty, lack of education, an increasing number of patients, and on the other hand, the demographic and epidemiological transitions; the insufficient number of rheumatologists and concentration of the workforce in large cities; an insufficient budget for education and research; and the backlog of accumulated problems characteristic of underdeveloped societies [2]. In consequence, RA is not yet considered a public health priority for the national health care systems, Therefore, explaining why only 56 % of RA patients had access to full medical health coverage (thus often excluding biologics), only 58 % had more than 7 years of education, and 58 % were of a low/low-middle socioeconomic status [3].

Previous efforts has been made to overcome this situation including: the identification of barriers to health care [4], promoting access to a prompt diagnosis and treatment, developing algorithms according these realities [5], educating patients and doctors [6], or estimating the economic impact of the disease in the region [7] and, recently, setting the standards to harmonize the treatment provided to RA patients in LA, by developing a program focused in establish centers of excellence that would offer to RA patients, a better care [8].

Additionally, technological progress, drug development and diagnostic capabilities, advanced in the past two decades has resulted in major improvements in health outcomes for RA patients in developed countries, but the cost of these innovations including joint prostheses, access to biological medications and the lack of trained health personnel to deliver the specialized care, still puts these out of the reach of people with RA in most low and middle income countries. Education and training of health professionals also remains an unmet key priority [2].

The Pan American League of Associations for Rheumatology (PANLAR) is an umbrella organization of Rheumatology National Societies in the American Continent, encompassing 21 countries, of those, 19 nations are located in the LA and Caribbean region. PANLAR seeks the following main goals [9]:

- Foster cooperation between different countries through existing national societies, organizations and colleges of Rheumatology and cooperating extensively with international organizations such as: EULAR, APLAR, AFLAR and ILAR.
- Promote the educational activities and organize in conjunction with the national societies the Pan American Congress of Rheumatology, as well as to promote national or regional meetings and encourage publication and dissemination of the scientific proceedings of meetings.
- Promote the educational activities and research of the PANLAR study groups.
- Develop new channels of communication among the member societies and stimulate scientific research into all aspects of the aims of PANLAR.

With these goals in mind and with the main objective focused on the revision and updating of the current challenges, difficulties and barriers faced by LA rheumatologists when treating RA and to find potential solutions (leverage points) to overcome the identified barriers, PANLAR is presenting in this supplement of Clinical Rheumatology nine relevant, challenging, and updated issues unique to LA region:

- Social and economic impact of RA and its relationship with health priorities.
- The path in seeking medical care of patients with RA.
- The importance of early diagnosis. Challenges and solutions.
- Proper management of RA. What the guidelines recommend?
- Access to optimal treatment. Current situation.
- Education of patients with RA.
- The training of allied health professionals.
- The model of comprehensive care.
- Challenges in LA for the implementation of an ideal model of health care center in patients with RA: are we ready to implement?

Each chapter has been developed by a Latin-American leading expert in the field in association with a group of experienced rheumatologists as coworkers, encompassing nine different countries in the region, thus reflecting the real challenges and opportunities for diagnosis and treatment of RA in LA. This review helps us to increase and improve the knowledge and approaches to this health condition in the region, thus promoting equity, quality, and efficiency of RA healthcare. The review includes not only the burden of the condition in LA but also its social, educational, and financial related aspects, furthermore, patient access to care, training of human resources, and novel approaches to models of care are analyzed.

Due to the current and future impact from RA in developing settings, health care systems need to develop coherent policies and strategies for dealing with this burden. Further epidemiological and health economics research is urgently needed [10] in this area and to better inform policy for dealing with RA in LA, to encourage multiple stakeholders (including funders, insurers, policy makers, educators, researchers, consumers, and health care givers) to co-operatively develop and implement efficient models of care to manage RA in developing countries.
